# Advanced electrochemical detection and profiling of the antihypertensive drug atenolol *via* a SPION-activated carbon nanocomposite interface

**DOI:** 10.1039/d5na00314h

**Published:** 2025-06-09

**Authors:** Ananya S. Agnihotri, Nidhin M

**Affiliations:** a Department of Chemistry, CHRIST (Deemed to be University) Bengaluru 560029 India nidhin.m@christuniversity.in

## Abstract

This study reports the synthesis of superparamagnetic iron oxide nanoparticles (SPIONs), activated carbon (AC) and SPION-AC nanocomposites using a simple hydrothermal method. Characterization of the synthesized materials includes dynamic light scattering, X-ray diffraction, field emission scanning electron microscopy, high resolution transmission electron microscopy, and vibrating sample magnetometry, along with electrochemical characterization studies such as electrochemical impedance spectroscopy. Among the SPION-AC nanocomposites, SPION-15%AC was employed to modify a glassy carbon electrode (GCE). The synergistic interaction between SPION and AC significantly enhanced the electrochemical properties of the system, leading to the development of a highly efficient platform for the detection of the antihypertensive drug atenolol (ATN) in commercial tablet samples. The sensor demonstrated excellent performance, with a linear detection range from 1.21 μM to 285 μM. With a low detection limit (LOD) of 0.401 μM, the sensor offers precise quantification of ATN, making it a promising tool for improving patient care. High selectivity, reproducibility, and excellent recovery in complex pharmaceutical matrices further highlight the potential of this sensor for biomedical and clinical applications.

## Abstract 2

Graphical illustration depicting the synthesis process of SPIONs and the SPION-AC nanocomposite.

## Graphical abstract

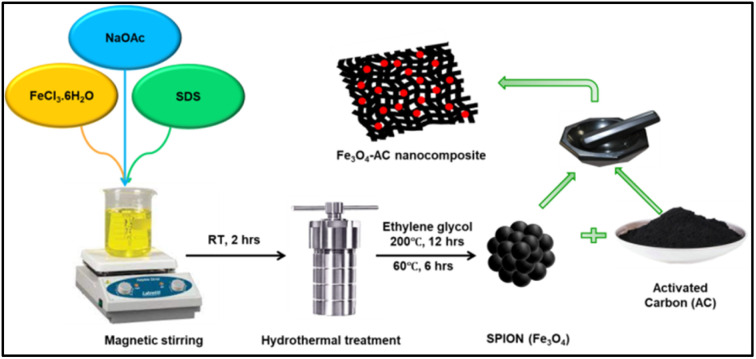

## 1. Introduction

**Fig. 1:**
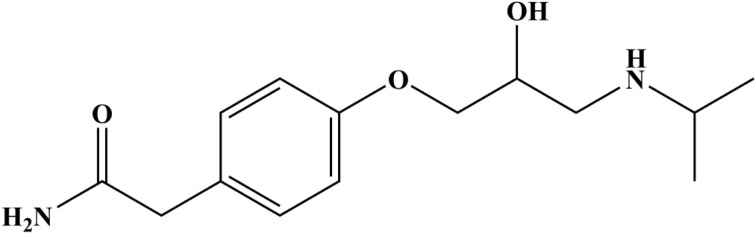
Chemical structure of atenolol.

β-Blockers are among the most commonly prescribed pharmaceutical drugs for managing high blood pressure and treating glaucoma.^1,2^ Atenolol (ATN), a cardioselective β1-blocker, is chemically known as 4-(2-hydroxy-3-isopropylaminopropoxy)-phenylacetamide (Fig. 1). It is particularly effective in treating angina pectoris, hypertension, cardiac arrhythmia, tremors, alcohol withdrawal, hyperthyroidism and migraine prophylaxis.^3^ ATN can be administered either orally or *via* injection. ATN exhibits low lipid solubility, which limits its ability to cross the blood–brain barrier.^4^ Its gastrointestinal absorption is relatively limited, resulting in an oral bioavailability of approximately 50%. Once absorbed, only a small fraction of ATN undergoes metabolism, with the majority being eliminated *via* the urine. In hypertensive patients, pharmacodynamic studies reveal that ATN can lower both systolic and diastolic blood pressure by around 15%. Typically, a dose of 100 to 200 mg reaches its peak efficacy on the third day of therapy. Compared to other β-blockers, ATN tends to reduce blood pressure largely through a decrease in heart rate (by 15–20%) and a reduction in cardiac output (by 20%), providing a consistent antihypertensive effect.^5,6^ However, excessive doses can lead to several adverse effects, including wheezing, hypotension, lethargy, bronchospasm, bradycardia, congestive heart failure, reduced cardiac output, cardiogenic shock, respiratory issues, hypoglycemia, and even sinus pause.^7^ Due to the toxicity and narrow therapeutic window of β-blockers, developing precise, sensitive, and selective detection methods for ATN is a priority in pharmaceutical research.

Several analytical methods have been utilized for ATN detection, including high-performance liquid chromatography,^8,9^ spectrofluorimetry,^10,11^ electrophoresis,^12,13^ voltammetry,^6,14^ diffuse reflectance spectroscopy,^15^ spectrophotometry,^16^ potentiometry,^17^ titrimetry,^18^ and gas chromatography.^19,20^ However, chromatographic techniques, while effective, are often complex, costly, time-consuming, and typically require sample extraction. Spectroscopic, potentiometric, and titrimetric methods, though useful, tend to lack the required sensitivity and are labor-intensive. Voltammetry, in contrast, is gaining popularity as an alternative for precise ATN detection due to its simplicity, rapid response, cost-effectiveness, and adaptability for various sample matrices, such as tablets,^21^ syrups,^22^ creams,^23^ shampoos^23^ and biological specimens.^24^ Studies exploring voltammetric approaches have tested various electrodes for ATN detection, including zinc oxide decorated graphene oxide composite modified glassy carbon electrode (GCE),^3^ mobile crystalline material-41 grafted with 3-aminopropyl groups on carbon paste electrode (MCM-41-*n*PrNH_2_/CPE),^25^ magnesium oxide nanoplatelet-modified screen-printed electrode,^26^ C_60_-modified GCE,^27^ Patton–Reeders reagent electropolymerized onto pencil graphite electrode (PR/PGE),^6^ gold nanoparticles/multiwalled carbon nanotubes modified GCE,^4,28^ CoFe_2_O_4_/graphene magnetic nanocomposite/CPE^29^ and several others. Among these modified carbon electrodes, the GCE is the best choice for the electrochemical detection of ATN due to its excellent chemical stability, low porosity, large surface area, strong electrical conductivity, broad potential window and simple electrode modification prospects. To maximize these benefits, we aim to improve the properties of the GCE by modifying it with tailor made nanomaterials in our sensor development. This strategy emphasizes the stability of the nanomaterial modifications while maintaining high sensitivity to ensure accurate detection.

Metal oxide nanoparticles such as CaO,^30^ SeO_2_,^31^ and mixed metal oxide nanoparticles composited with carbonaceous matrices^32^ play a crucial role in the electrochemical sensing of a wide range of analytes due to their excellent conductivity, large surface area and catalytic properties.^33^ These features enhance the sensitivity and accuracy of sensors, enabling precise detection even at low drug concentrations. Superparamagnetic iron oxide nanoparticles (SPIONs), in particular, are highly valued in electrochemical sensing of pharmaceutical drugs due to their unique combination of magnetic and conductive properties.^34^ Their superparamagnetic behavior enables precise control and immobilization within sensor systems, enhancing both sensor stability and reproducibility in drug detection. The large surface-to-volume ratio of SPIONs facilitates a greater loading of active sites, while their excellent electrical conductivity promotes faster electron transfer, significantly boosting sensor sensitivity.^35^ This heightened sensitivity is critical for the accurate detection of low drug concentrations, making SPIONs an ideal choice for developing advanced electrochemical sensors that can reliably monitor pharmaceuticals. Their biocompatibility and ease of surface modification further support the selective and reliable detection of specific target molecules. Their versatility and efficiency position SPIONs at the forefront of cutting-edge pharmaceutical analysis.

Activated carbon (AC) is yet another promising material in the electrochemical sensing of antihypertensive drugs, primarily due to its exceptional surface area, porous structure and excellent conductivity. These properties enhance the adsorption and catalytic efficiency of the sensor, making it possible to detect pharmaceutical drugs at very low concentrations.^36^ The extensive surface area and high porosity of AC provide numerous electro-active sites, facilitating increased interactions with target drug molecules and thus improving detection sensitivity and response time. Furthermore, its conductivity aids in efficient electron transfer, a primary factor in achieving accurate electrochemical signals. AC not only improves the sensor performance but also contributes to stability and cost-effectiveness, making it an ideal material for developing robust, high sensitivity sensors for antihypertensive drugs.

This study is dedicated to demonstrating the effective modification of the GCE using a SPION-AC nanocomposite for the advanced electrochemical detection and profiling of ATN within complex pharmaceutical formulations. By harnessing the unique advantages of the GCE alongside the combined stability and functionality of SPIONs and activated carbon in the SPION-AC nanocomposite, this research aims to progress electrochemical sensing technology for accurate and efficient ATN detection. In this work, the authors report for the first time the use of a SPION-AC nanocomposite for the electrochemical determination of ATN in pharmaceutical formulations. The results reveal the significant potential of SPION-AC/GCE for practical, reliable drug monitoring. The porous structure of SPION-AC nanocomposite not only enhances the ATN adsorption on the electrode surface but also enables rapid electron transfer, resulting in exceptional sensitivity and selectivity. The synergistic effect of SPIONs and AC contributes to the improved conductivity, stability and availability of electroactive sites, positioning SPION-AC/GCE as a promising approach for advanced ATN detection in pharmaceutical samples.

## 2. Experimental section

### 2.1. Materials and methods

Ferric chloride hexahydrate (FeCl_3_ × 6H_2_O) (98.0%), ethylene glycol (EG) (99.0%), sodium acetate (NaOAc) (99.0%), lauryl sodium sulfate (SDS), activated carbon, acetic acid (99.8%), boric acid (99.5%), phosphoric acid (85%), potassium ferricyanide (97%) and potassium chloride were procured from S D Fine chemicals. Nafion, used as the binding material in the fabrication of the ATN sensor, was procured from Sigma-Aldrich. ATN was a gift sample. Britton–Robinson buffer (BR buffer) was prepared by mixing equal volumes of phosphoric acid, acetic acid and boric acid. The desired pH was achieved by adding 0.2 M NaOH to the BR buffer solution. All the chemicals procured were of analytical grade and were used directly without any further purification.

### 2.2. Synthesis of SPIONs (Fe_3_O_4_ nanoparticles) *via* hydrothermal route

SPIONs were synthesized using a hydrothermal method as described in a previous report.^37^ In a typical synthesis, 2.16 g of FeCl_3_·6H_2_O was dissolved in 80 mL of EG and stirred for 1 h, resulting in a clear yellow solution. Following this, 3.6 g of NaOAc, and 0.5 g of SDS were added, and the mixture was stirred vigorously for 1 h. The final solution was then placed in a Teflon-lined stainless steel autoclave with a 100 mL capacity, sealed, and heated at 200 °C for 12 h before cooling to room temperature. The black precipitate was collected, thoroughly washed with ethanol and deionised water, and then dried under vacuum for 6 h at 60 °C.

### 2.3. Reactivation of AC and preparation of SPION-AC nanocomposites

**Scheme 1:**
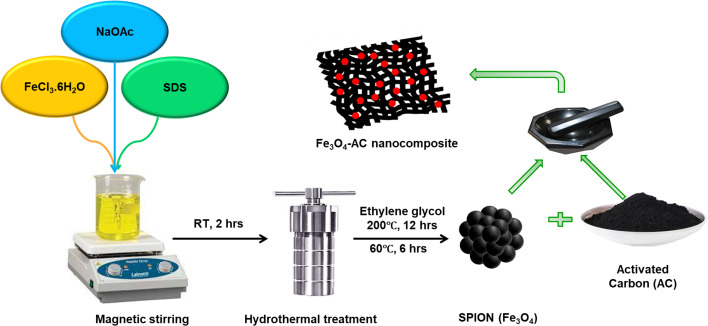
Graphical illustration for the synthetic protocol of SPIONs and SPION-AC nanocomposites.

To enhance the functionality of the commercially obtained AC, it was reactivated using an acid treatment process. A precise amount of AC was placed in a clean round-bottom flask, followed by the addition of concentrated 75% HNO_3_. This mixture was stirred at a moderate speed for 48 h 60 °C to facilitate reactivation. The reactivated AC was then collected, vacuum dried, and stored in an airtight container for further applications. For the preparation of SPION-AC nanocomposites, a defined quantity of SPIONs was combined with varying proportions of AC (1%, 5%, 10%, and 15%) in a clean agate mortar. The components were blended thoroughly for approximately 45 minutes to ensure complete homogenization. The resultant nanocomposites were individually collected and stored in airtight containers for future use, labelled as SPION-1% AC, SPION-5% AC, SPION-10% AC, and SPION-15% AC, respectively. This systematic preparation approach ensured precise composition and uniform distribution within each nanocomposite, providing a robust sensing platform for the electrochemical determination of ATN. The schematic representation of the synthetic protocol has been provided in Scheme 1.

### 2.4. Fabrication of a SPION-AC/GCE sensor for precise electrochemical profiling of ATN

Prior to electrode preparation, the GCE was meticulously polished with alumina microparticles on a polishing pad until a smooth, mirror-like surface was achieved. Following polishing, the GCE was sonicated sequentially in water and ethanol to remove any residual particulate contaminants, and subsequently air-dried at ambient temperature. For separate suspensions, 2 mg each of SPIONs, AC, and SPIONs with 1%, 5%, 10%, and 15% AC were dispersed in 1 mL of Nafion solution. These mixtures were then ultrasonicated for 30 minutes to ensure homogeneity. A 15 μL aliquot of each suspension was drop-cast onto the GCE surface to form SPION/GCE, AC/GCE, SPION-1%AC/GCE, SPION-5% AC/GCE, SPION-10%AC/GCE, and SPION-15%AC/GCE.

### 2.5. Instrumentation

The crystal structures of the synthesized SPION, AC, and SPION-AC nanocomposites were examined using a Rigaku Smart Lab X-ray diffractometer with Cu Kα radiation at a wavelength of 1.5406 Å, covering a 2*θ* range of 10° to 80°. Morphological analysis was conducted through field emission scanning electron microscopy (FESEM) with a JEOL 6390LA system operating up to 30 kV and transmission electron microscopy (TEM) using a JEM-2100. Elemental composition and distribution were investigated through energy-dispersive X-ray (EDX) analysis and elemental mapping. The particle size and surface charge (zeta potential) were measured with a Malvern Zetasizer (ZEN3600) at room temperature. Magnetic properties of the SPION-AC nanocomposite were evaluated *via* vibrating sample magnetometry (VSM).

Electrochemical properties of SPION, AC, SPION-1%AC, SPION-5%AC, SPION-10%AC, and SPION-15%AC were evaluated by cyclic voltammetry (CV) and electrochemical impedance spectroscopy (EIS). Working electrodes included a GCE, SPION/GCE, AC/GCE, SPION-1%AC/GCE, SPION-1%AC/GCE, SPION-5%AC/GCE, SPION-10%AC/GCE and SPION-15%AC/GCE, with a saturated calomel reference electrode and platinum foil as the counter electrode. CV and EIS measurements were performed using a CH instruments electrochemical workstation (model CH1608), while differential pulse voltammetry (DPV) was conducted with the CH1609 model in a three-electrode setup. A 0.04 M BR buffer solution (pH 10) was used as the electrolyte in ATN sensing experiments. EIS studies took place in a solution containing 1 mM K_3_[Fe(CN)_6_] and 0.1 M KCl over a frequency range of 1 Hz to 100 kHz with a 0.005 A amplitude.

### 2.6. Electrochemical detection of ATN

For the electrochemical detection of ATN, CV measurements were carried out with the SPION-15%AC/GCE as the working electrode, sweeping the potential from 0.6 V to 1.2 V at a scan rate of 0.05 V s^−1^. To enable a comparative analysis, these experiments were repeated with each modified GCE as well as the unmodified, bare GCE, thereby allowing an evaluation of each electrode's sensitivity toward ATN detection. Additionally, differential pulse voltammetry (DPV) was employed to quantitatively assess ATN, using the same potential range (0.5 V to 1.2 V) on the modified electrode. A calibration curve was established from the oxidation peak current of ATN at 0.98 V *vs.* SCE, facilitating accurate quantification.

### 2.7. Preparation of ATN stock solution and real samples

To prepare the ATN stock solution, an ATN reference sample was precisely weighed to yield a concentration of 25 mM and dissolved in a 1 : 1 mixture of acetone and water. This solution was then brought to volume in a 25 mL volumetric flask. Further dilutions were made using acetone and Britton–Robinson (BR) buffer to obtain the desired ATN working standard solutions. Fresh working solutions were prepared immediately prior to ATN sensing to maintain accuracy and reliability in the results.

To analyze ATN in commercial tablets, 10 tablets were finely ground with a mortar and pestle. A known amount of this powder, equivalent to 25 mM ATN, was dissolved in a 100 mL flask with a 50 : 50 acetone–water mixture. The solution was sonicated for 20 minutes to ensure complete dissolution, and then filtered through standard filter paper. The resulting clear supernatant was used to prepare working solutions by diluting with a supporting electrolyte. Recovery tests were conducted to verify the accuracy of the proposed method and check for interference from excipients. Finally, the ATN content was quantified using a calibration curve or regression analysis.

## 3. Results and discussion

### 3.1. Structural and morphological characterization

**Fig. 2:**
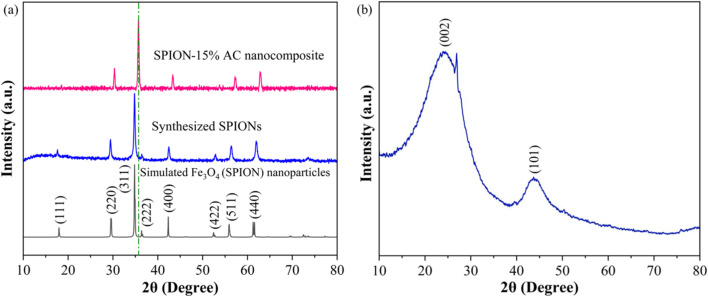
(a) XRD patterns of the synthesized SPIONs and SPION-15% AC nanocomposites, and the simulated XRD pattern of SPIONs for comparison. (b) X-ray diffractogram highlighting the structural features of AC.

The XRD technique serves as a powerful tool for investigating the crystalline structure, phase purity, and average crystallite size of synthesized nanomaterials and their corresponding nanocomposites. In this study, XRD analysis was employed to characterize the structural properties of SPIONs and SPION-AC nanocomposites, providing insights into phase composition and crystallite size, which are critical for understanding their functional performance. The diffraction pattern in Fig. 2(a) reveals prominent, well-defined peaks for SPIONs (Fe_3_O_4_), and the SPION-AC nanocomposite, indicating a high degree of crystallinity across these materials. The SPIONs exhibit eight distinct peaks at 2*θ* values of 18.9°, 30.1°, 36.4°, 37.8°, 43.4°, 54.2°, 57.5°, and 62.5°, corresponding to the (111), (220), (311), (222), (400), (422), (511), and (440) lattice planes, characteristic of the cubic inverse spinel structure associated with the *Fd*3̄*m* space group.^37^ This pattern confirms the well-defined crystallinity and stable structure of SPIONs. For activated carbon, the XRD pattern shown in Fig. 2(b) displays two broad peaks near 2*θ* values of approximately 24° and 42°, corresponding to the (002) and (100) planes. The broadness of these peaks is indicative of an amorphous structure of the activated carbon, suggesting a lack of long-range atomic order typically observed in highly crystalline materials. In the SPION-AC nanocomposite (Fig. 2(a)), the XRD pattern shows a slight shift in the 2*θ* positions of certain peaks, indicating an interaction between the SPIONs and the activated carbon matrix, leading to the formation of the composite structure. Notably, two of the characteristic peaks corresponding to the (111) and (422) planes from the SPIONs are absent in the SPION-AC pattern, suggesting either a partial masking of the crystalline SPION structure due to the AC matrix or a possible reduction in crystalline ordering upon composite formation. This reduction in peak intensity and the disappearance of specific peaks can imply an altered or diminished crystallinity of the SPIONs when integrated into the composite, possibly due to interactions at the nanoscale that influence the structural properties of the SPION-AC composite material. The average crystallite size of the SPIONs and the SPION-AC nanocomposite was calculated using the Scherrer equation (eqn (1)):1
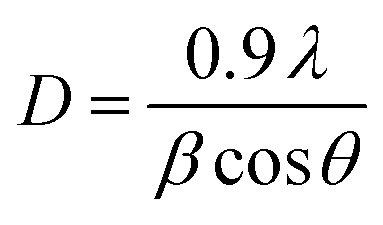
where *D* is the crystallite size, *λ* is the wavelength of the X-ray source, *β* is the full width at half maximum of the diffraction peaks in radians, 0.9 is the shape factor and *θ* is the Bragg angle. The crystallite sizes for all the observed peaks were calculated, revealing an average crystallite size of 6.83 nm for the SPIONs and 13.68 nm for the SPION-AC nanocomposite. In addition, the absence of additional peaks suggests high purity and phase uniformity of the synthesized SPIONs, AC and SPION-AC nanocomposites.

**Fig. 3:**
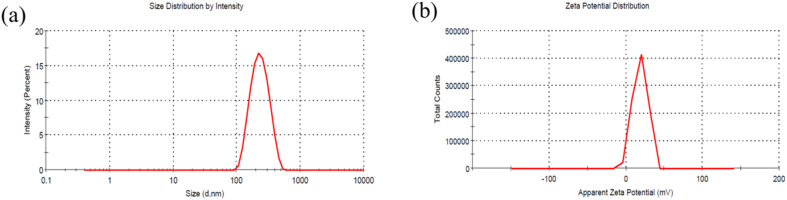
(a) Size *versus* intensity plot showing the hydrodynamic diameter and (b) zeta potential plot of synthesized SPIONs.

Dynamic light scattering (DLS) is a key technique for measuring the particle size and hydrodynamic diameter of SPIONs, providing critical insights into their size distribution and colloidal stability. DLS offers precise, non-invasive and rapid characterization by measuring the scattering of light caused by the Brownian movement of SPIONs in a suspension. Fig. 3(a) illustrates the particle size distribution of SPIONs, while Fig. 3(b) depicts their zeta potential plot. The SPIONs exhibit a hydrodynamic diameter of 182.1 nm and a polydispersity index of 0.2, indicative of their monodisperse nature. The narrow, bell shaped curve observed in the size distribution plot underscores the synthesis of uniformly sized SPIONs. Furthermore, the presence of a distinct monomodal peak observed in the intensity *versus* size plot suggests the formation of spherical SPIONs. The zeta potential, a critical parameter for evaluating colloidal stability, reflects the surface charge of nanoparticles. It quantifies the electrostatic repulsion between similarly charged SPIONs, with higher values signifying enhanced repulsion and greater stability in various environments. Fig. 3(b) presents a zeta potential value of 21.6 mV for the SPIONs, affirming their stability. The zeta potential value suggests a strong resistance to aggregation, ensuring their effective dispersion and robustness in diverse applications. The combination of monodispersity, uniform morphology, and high stability positions these SPIONs as promising candidates for advanced electrochemical profiling of ATN.

**Fig. 4:**
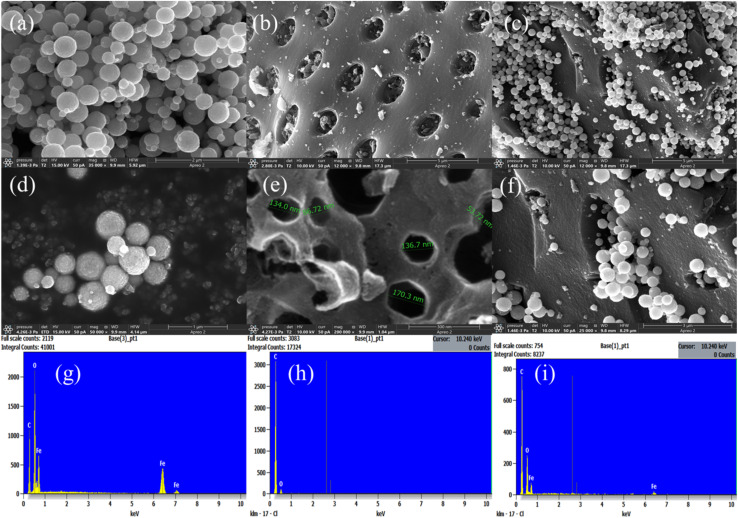
FESEM images showcasing the morphological features of (a and d) SPIONs, (b and e) AC, and (c and f) the SPION-AC nanocomposite, along with their corresponding EDAX spectra in panels (g, h and i).

The morphology of the synthesized SPIONs, AC, and SPION-AC nanocomposites was meticulously analyzed using FESEM micrographs, revealing significant structural insights. The morphology of SPIONs, shown in Fig. 4(a and d), demonstrates their distinctive spherical nanocluster arrangement. These nanoclusters exhibit rough surfaces, with each cluster composed of numerous grain-like SPIONs, as confirmed by Fig. 4(d) and further supported by HRTEM analysis. The clustering of SPIONs is attributed to the strong magnetic interactions among them, as corroborated by VSM analysis. Fig. 4(b and e) highlight the porous architecture of AC, characterized by uniformly sized and equidistant pores, as evidenced in Fig. 4(c). A detailed examination reveals that the majority of pore diameters range between 134 nm and 137 nm, with a few larger pores measuring approximately 171 nm. The average pore wall thickness is calculated to be 53.7 nm, forming a robust porous structure that facilitates efficient channels for rapid electron transfer, particularly advantageous for electrochemical detection of ATN. The successful synthesis of the SPION-AC nanocomposite is clearly demonstrated in Fig. 4(c and f). The micrographs show that SPIONs are uniformly distributed across the AC surface, effectively filling the empty spaces within the AC pores. This uniform dispersion ensures a strong integration between the SPION and AC components, enhancing the composite's functional properties. Additionally, the elemental composition of SPIONs, AC, and the SPION-AC nanocomposite was verified through EDAX spectra, shown in Fig. 4(g, h and i). The detection of Fe, C, and O elements in the SPION-AC composite strongly supports the successful formation of this nanocomposite. This precise structural integration and composition further validate its potential for advanced applications, including efficient electrochemical systems.

**Fig. 5:**
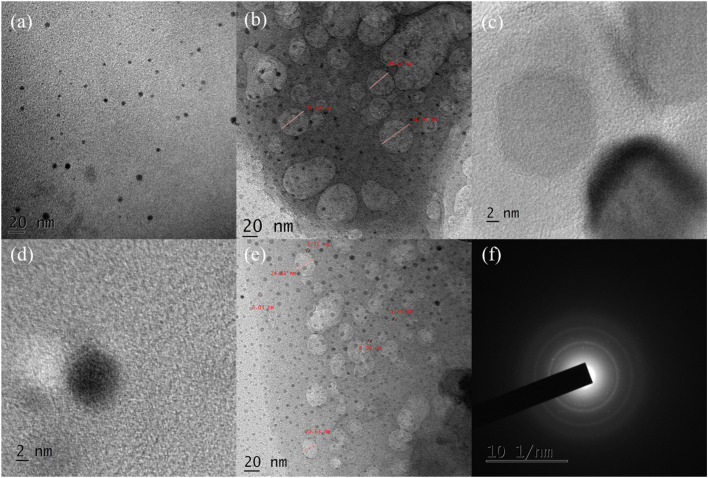
(a and d) HRTEM images of SPIONs, (b, c and e) morphological features of the SPION-AC nanocomposite, and (f) the SAED pattern of the synthesized SPION-AC nanocomposite.

Fig. 5 presents the TEM and HRTEM images of SPIONs and the SPION-AC nanocomposite, offering detailed insights into their unique morphological characteristics. The TEM and HRTEM images of SPIONs in Fig. 5(a and b) reveal a distinct spherical morphology, with individual nanoparticles appearing well-dispersed and exhibiting minimal aggregation. This dispersion underscores the efficiency of the synthesis process in mitigating nanoparticle clustering. Within the clustered SPION nanospheres, each nanoparticle displays a clearly defined grain boundary, signifying a high degree of uniformity. The particle size, ranging from 4 nm to 9 nm, aligns closely with the crystallite size calculated from XRD analysis (6.83 nm). Furthermore, the distinct lattice fringe spacing of 0.25 nm corresponds to the (311) plane of the face-centered cubic (fcc) inverse spinel structure of magnetite, confirming the crystalline nature of the nanoparticles. Fig. 5(b, c and e) provide a comprehensive view of the morphological characteristics of the SPION-AC nanocomposite. Fig. 5(b and e) illustrate that the nanocomposite comprises activated carbon (AC) sheets adorned with numerous pores, with SPIONs uniformly distributed across the AC surface. Remarkably, the size and morphology of the SPIONs remain intact even after integrating into the SPION-AC nanocomposite, emphasizing the stability of the nanoparticles during the synthesis process. The HRTEM image in Fig. 5(c) reveals the spherical geometry of both the SPIONs and the pores within the AC matrix. This image further highlights the presence of highly ordered lattice fringes within the SPIONs, indicative of their crystalline structure. The clearly visible lattice fringes confirm that the crystalline integrity of the SPIONs is preserved in the composite. Moreover, Fig. 5(f) depicts the selected area electron diffraction (SAED) pattern, characterized by a combination of bright spots and diffused rings. This pattern serves as evidence of the polycrystalline nature of the SPION-AC nanocomposite, suggesting that the material retains both crystalline order and structural heterogeneity.

The combination of uniformly dispersed nanoparticles, intact morphological features, and a well-defined polycrystalline structure underscores the efficacy of the synthesis process in producing a robust and structurally coherent SPION-AC nanocomposite.

**Fig. 6:**
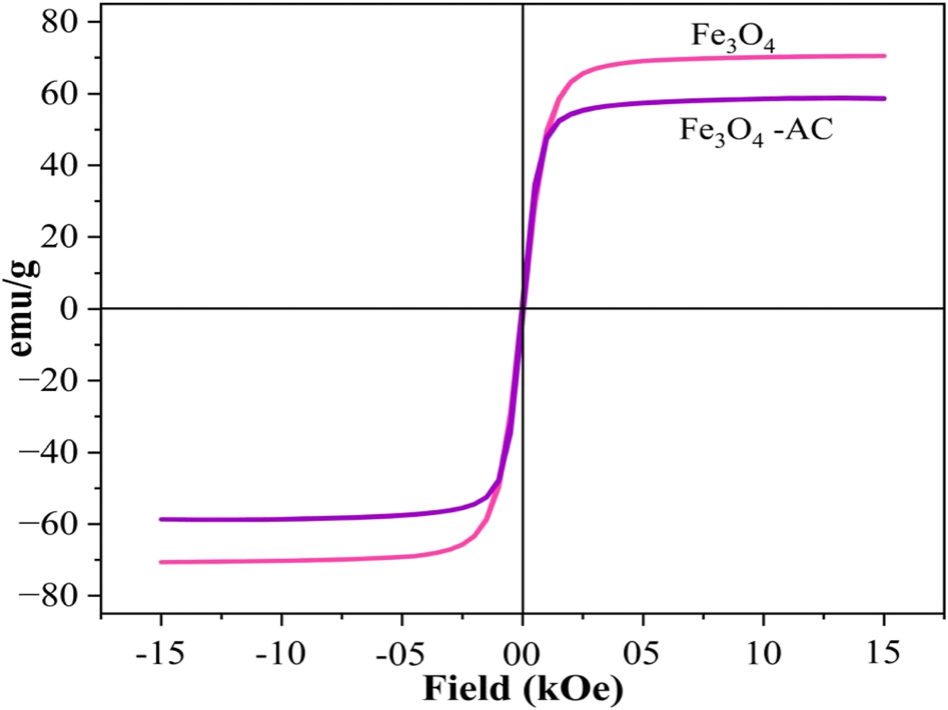
VSM analysis of the synthesized SPIONs and SPION-AC nanocomposites at room temperature.

The magnetic behavior of the synthesized SPIONs was analyzed using Vibrating Sample Magnetometry (VSM). The resulting magnetization curve, as shown in Fig. 6, exhibited a distinct S-shaped hysteresis loop, confirming the superparamagnetic nature of the nanoparticles. This behaviour is characterized by the absence of remanence and coercivity, indicating that the SPIONs lose their magnetization once the external magnetic field is removed. The saturation magnetization (*M*_S_) was measured at an impressive 69.89 emu g^−1^, reflecting the strong magnetic response of the nanoparticles. Such properties are particularly advantageous for applications in electrochemical sensing, where superparamagnetism allows nanoparticles to be effectively manipulated by external magnetic fields without retaining permanent magnetization. However, upon forming the SPION-AC nanocomposite, a noticeable reduction in saturation magnetization was observed, with the value decreasing to 59.36 emu g^−1^. This decline is primarily due to the incorporation of activated carbon (AC) in the composite, which dilutes the magnetic contribution of SPIONs. Despite this reduction, the superparamagnetic nature of the material remains intact, ensuring that the SPION-AC nanocomposite retains its responsiveness to external magnetic fields while benefiting from the structural and functional enhancements provided by the AC.

### 3.2. Electrochemical behavior of SPION-AC nanocomposites

**Fig. 7:**
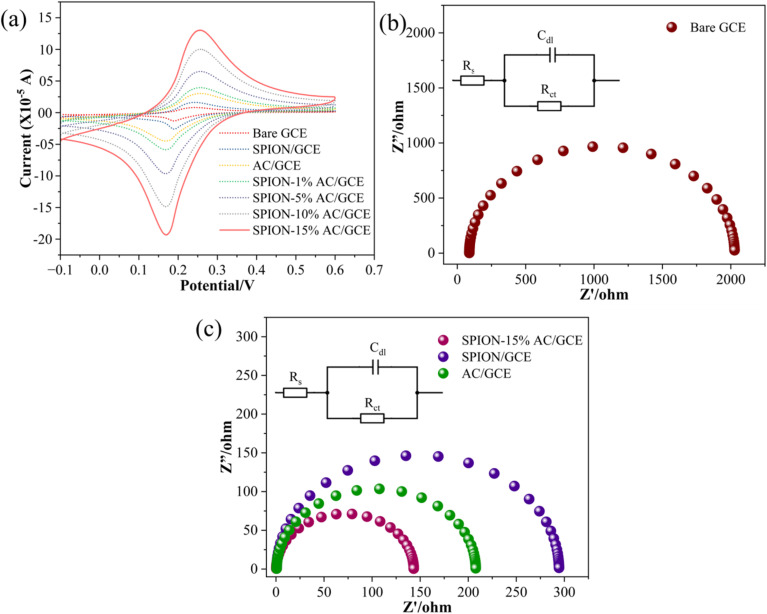
(a) Superimposed CV profiles of the bare GCE and modified electrodes; Nyquist plots of (b) the bare GCE and (c) modified electrodes in 0.1 M KCl containing 1 mM hexacyanoferrate redox system.

To evaluate the electrochemical behavior of the bare glassy carbon electrode (GCE) and its modified forms, CV experiments were performed. A typical experiment involved using a solution containing 1 mM K_3_[Fe(CN)_6_]^3−^/^4−^ and 0.1 M potassium chloride, with CV scans conducted at a scan rate of 0.05 V s^−1^, as shown in Fig. 7(a). The results revealed that the bare GCE exhibited a minimal current response, which progressively increased upon electrode modification in the following order: bare GCE < SPION < AC < SPION-1%AC < SPION-5%AC < SPION-10%AC < SPION-15%AC.The enhanced current response observed in electrodes modified with superparamagnetic iron oxide nanoparticles (SPIONs) and activated carbon (AC) can be attributed to the high surface area-to-volume ratio of SPIONs and the excellent electrical conductivity of AC. Furthermore, the SPION-AC composites demonstrated a significantly improved and broader current response due to the synergistic interaction between SPIONs and AC, which increased the electroactive surface area of the electrodes. To quantify this enhancement, the electroactive surface areas of the bare and modified electrodes were determined using the Randles–Sevcik equation (eqn (2)):^38^

2 *i*_p_ = 2.69 × 10^5^*AD*_o_^1/2^*n*^3/2^*ν*^1/2^*C*

where *A* is the electroactive surface area (cm^2^), *i*_p_ is the peak current (A), *D*_o_ is the diffusion constant in cm^2^ s^−1^, *n* is the number of electrons taking part in the redox reaction, *ν* is the scan rate (V s^−1^), and *C* is the concentration of the redox probe (mol cm^−3^) in the bulk of the solution. Based on this equation, the calculated electroactive surface areas were 0.068 cm^2^ for the bare GCE, 0.668 cm^2^ for SPION/GCE, 0.894 cm^2^ for AC/GCE, and 1.483 cm^2^ for SPION-15%AC/GCE, confirming the superior electrochemical performance of the modified electrodes.

The electrochemical performance of the modified electrodes is further substantiated by EIS analysis, which offers critical insights into charge transfer resistance, capacitance, and diffusion processes. By applying an alternating current (AC) signal and measuring the frequency-dependent response of the system, EIS provides a comprehensive understanding of interfacial electron transfer dynamics. The impedance data are commonly represented using Nyquist and Bode plots, with Nyquist plots being particularly useful for visualizing the interplay between real and imaginary impedance components. In a typical Nyquist plot, the high-frequency region is characterized by a semicircle, while the low-frequency region appears as a linear segment with a slope of approximately 45°. The semicircular portion corresponds to the charge transfer process, with the diameter of the semicircle directly reflecting the charge transfer resistance (*R*_CT_), a key determinant of electron transfer kinetics. The linear segment at lower frequencies represents diffusion-controlled processes or mass transport within the system.^39^ The fitted curves, depicted in Fig. 7(b and c), are modeled using a Randles equivalent circuit, incorporating parameters such as solution resistance (*R*_S_), charge transfer resistance (*R*_CT_), and double-layer capacitance (*C*_dl_), as indicated in the inset. The *R*_CT_ value, derived from the semicircle diameter, quantifies the efficiency of electron transfer at the electrode–electrolyte interface. For the unmodified bare GCE, a high *R*_CT_ of 2024 Ω was observed (Fig. 7(b)), indicative of sluggish electron transfer kinetics. In contrast, significant reductions in *R*_CT_ were observed for the modified electrodes, with values of 294 Ω for SPION/GCE, 208 Ω for AC/GCE, and an impressively low 142 Ω for SPION-15%AC/GCE (Fig. 7(c)). The dramatic reduction in *R*_CT_ for SPION-15%AC/GCE highlights the synergistic interaction between SPIONs and activated carbon. The high surface area of SPIONs enhances active site availability, while the excellent electrical conductivity of AC facilitates efficient charge transport. Moreover, the porous architecture of the SPION-AC nanocomposite promotes rapid electron transfer by enabling seamless interaction between the electrode and electrolyte. Among all fabricated electrodes, SPION-15%AC/GCE exhibited the lowest *R*_CT_, confirming its superior electrochemical performance. This remarkable enhancement in electron transfer kinetics underscores the synergistic effects of SPIONs and activated carbon, demonstrating their combined potential to significantly improve the conductivity and charge transfer efficiency of the electrode.

**Fig. 8:**
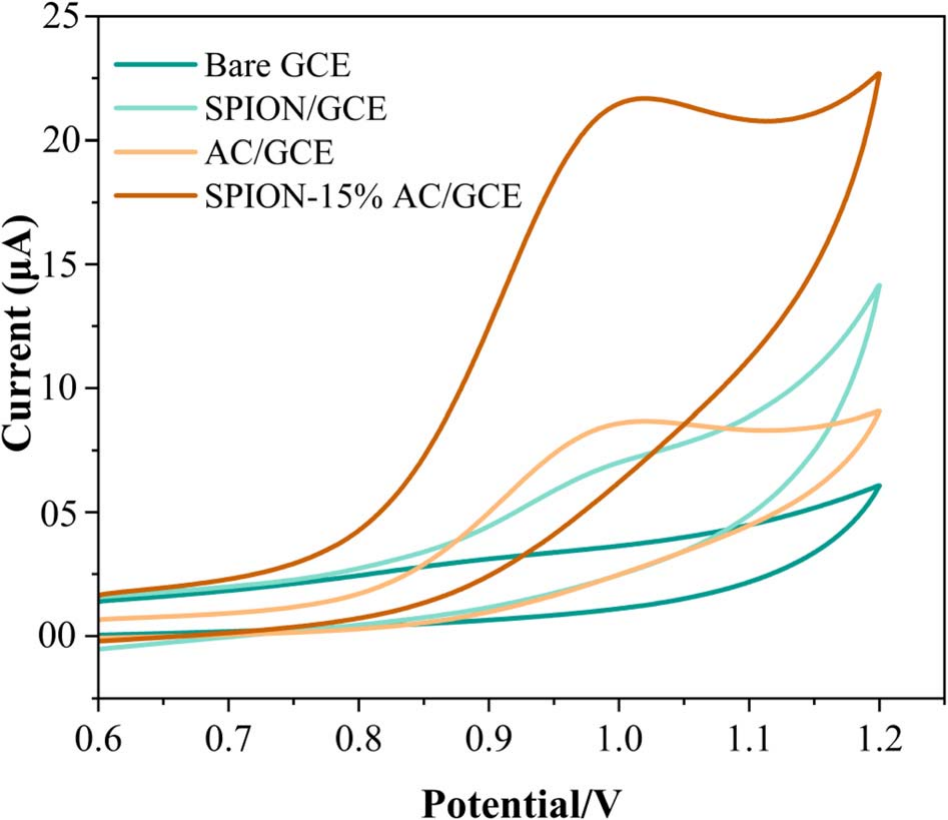
Superimposed cyclic voltammograms for the electrochemical detection of 80 μM ATN at 0.05 V s^−1^ in BR buffer solution of pH 10 at the bare GCE, SPION/GCE, AC/GCE, and SPION-15%AC/GCE surfaces.

The electrochemical behavior of ATN was investigated on a bare GCE and various modified electrodes using cyclic voltammetry in a pH 10 buffer solution, employing a scan rate of 0.05 V s^−1^ within the potential window of 0.6 V to 1.2 V, as shown in Fig. 8. The bare GCE did not exhibit any detectable oxidation peak for ATN, indicating its limited sensitivity and lack of catalytic activity. However, modifying the GCE with SPIONs resulted in a subtle oxidation peak at 0.97 V with a peak current of 6.456 μA, signifying a modest enhancement in the electrode's electrochemical activity. A more pronounced oxidation peak was observed with the AC-modified GCE (AC/GCE), where the current increased slightly to 8.557 μA while retaining the same peak potential. This improvement highlights the increased electroactive surface area and superior electrical conductivity of the AC-modified electrode.

The most significant performance enhancement was achieved with SPION-15%AC/GCE, which displayed an oxidation peak at 0.95 V, indicating a slight negative shift in potential. The faradaic current increased substantially to 21.591 μA, representing a dramatic improvement in the electrode's sensitivity toward ATN oxidation. This marked enhancement is attributed to the synergistic effects of SPIONs and AC, which together provide a highly conductive network and excellent catalytic properties, thereby facilitating faster and more efficient electron transfer. These findings were corroborated by EIS data, which demonstrated reduced charge transfer resistance for SPION-15%AC/GCE, further supporting its enhanced electrochemical performance. This indicates that the SPION-15%AC/GCE outperformed both the bare GCE and other modified electrodes in terms of sensitivity, peak current response, and catalytic efficiency, establishing it as the most effective platform for ATN detection and quantification. This superior performance underscores the pivotal role of optimized composite materials in advancing electrochemical sensor technology towards ATN detection.

**Fig. 9:**
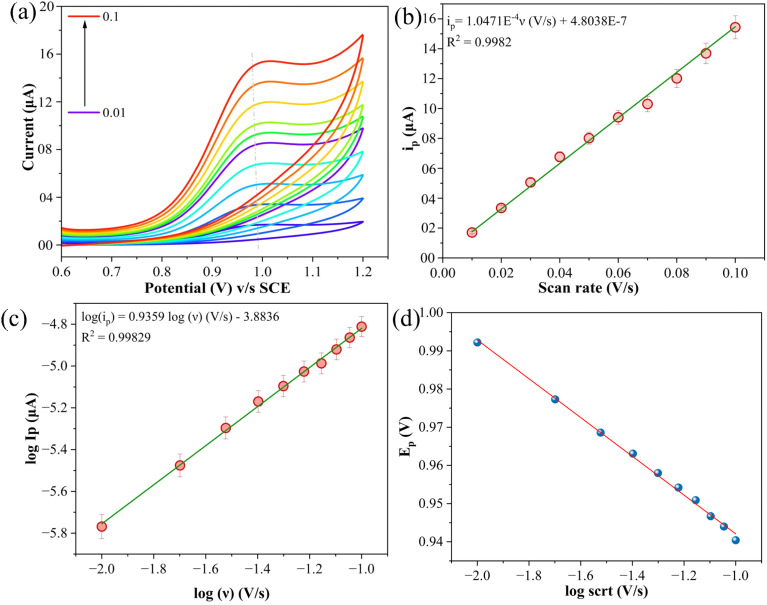
(a) Superimposed cyclic voltammograms for the electrochemical detection of 80 μM ATN in BR buffer solution (pH 10) at varying scan rates between 0.01 V s^−1^ to 0.1 V s^−1^, (b) a plot of scan rate *versus* ATN oxidation peak current, (c) a log–log plot of scan rate *versus* ATN oxidation peak current, and (d) linear plot of logarithm of scan rate *versus* peak potential.

The electrochemical oxidation of ATN was thoroughly examined using cyclic voltammetry to determine the number of electrons involved in the redox process. Fig. 9(a) displays the overlaid voltammograms recorded for the SPION-15%AC/GCE at varying scan rates, ranging from 0.01 V s^−1^ to 0.1 V s^−1^, in a pH 10 BR buffer solution. A key method for identifying the rate-determining step of a well-calibrated electrode involves analyzing the relationship between the anodic peak current and either the scan rate or the square root of the scan rate.^40^ In this case, the anodic peak current did not exhibit a proportional relationship with the square root of the scan rate (data not shown), suggesting that the process is not diffusion-controlled. Instead, a linear correlation between the anodic peak current and the scan rate (*i.e.*, *i*_pa_∝*ν*) was observed, as illustrated in Fig. 9(b). This behaviour unequivocally indicates that the electrochemical process is predominantly governed by surface adsorption rather than diffusion. A corresponding linear regression analysis corroborates this adsorption-controlled mechanism.

3 *i*_p_ = 1.0471 × 10^−4^*ν*(V s^−1^) + 4.8038 × 10^−7^ (*R*^2^ = 0.9982)

Additionally, a log–log plot of scan rate *versus* anodic peak current demonstrated a strong linear relationship (Fig. 9(c)), further validating the adsorption-dominated nature of the reaction. The resulting linear regression equation provides quantitative support for this observation.

4 log(*i*_p_) = 0.9359 log(*ν*)(V s^−1^) − 3.8836 (*R*^2^ = 0.9986)

The dependence of peak potential (*E*_p_) on the logarithm of scan rate, depicted in Fig. 9(d), is described using eqn (5):^41^

5 *E*_p_ = (*b*/2) log *ν* + constant

Here, *b* is calculated using the relation *b* = 2.303*RT*/*αnF* ∼0.18, where *α* represents the electron transfer coefficient, and the other terms retain their conventional meanings. From this, the value of *α* was determined to be 0.45. To further quantify the process, the number of electrons (*n*) involved in the oxidation of ATN on the SPION-15%AC/GCE surface was calculated using Bard and Faulkner's equation (eqn (6)),^42^ which relates the peak potential (*E*_p_) and the half-peak potential (*E*_p_/2).

6 *E*_p_ = [2.303*RT*/(1 − *α*)*nF*] log *ν* + *K*, *α* = 47.7/*E*_p_ − *E*_p/2_

**Scheme 2:**
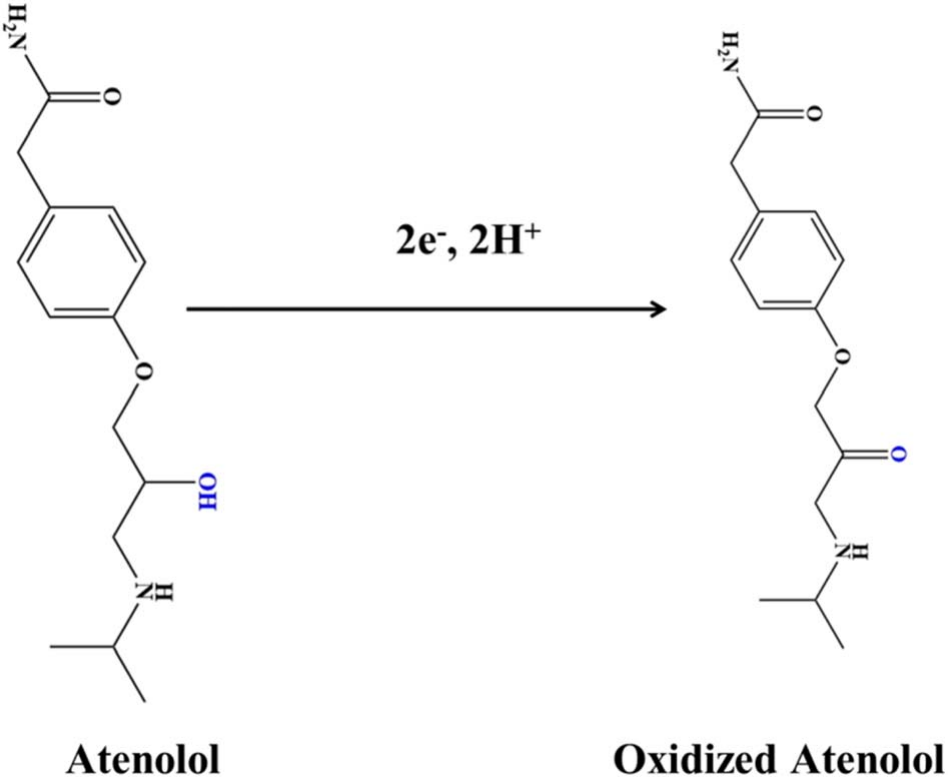
Plausible mechanism for the electrochemical sensing of ATN at the SPION-15%AC/GCE surface.

Based on the substituted experimental values, *n* was calculated to be approximately 1.96, rounding to 2. This result suggests a two-electron transfer mechanism, as illustrated in Scheme 2, which outlines the proposed oxidation pathway for ATN. The mechanism for the electrochemical oxidation of ATN can be elucidated as follows: the electrochemical oxidation mechanism of atenolol on the SPION-15%AC/GCE is governed by a surface-confined redox process involving the transformation of its benzylic hydroxyl group. As illustrated in the provided scheme, atenolol undergoes a two-electron, two-proton oxidation, wherein the benzylic –CH(OH)– moiety is converted into a corresponding ketone –C(=O)– functionality. The adsorption of atenolol onto the high-surface-area SPION-15%AC/GCE matrix is facilitated by π–π stacking interactions between the aromatic ring and the carbon surface, as well as potential hydrogen bonding and coordination with surface hydroxyl groups and Fe^3+^ centers on SPIONs. The conductive and redox-active nature of the SPION synergistically enhances electron transfer kinetics, while the AC ensures efficient analyte pre-concentration through strong adsorptive interactions. This concerted interaction not only lowers the oxidation potential but also amplifies the electrochemical signal, thus enabling sensitive and selective detection. The transformation of the hydroxyl group into a ketone, as shown by the structural shift, confirms a proton-coupled electron transfer mechanism, highlighting the utility of SPION-15%AC composites in advanced electrochemical sensing platforms.

**Fig. 10:**
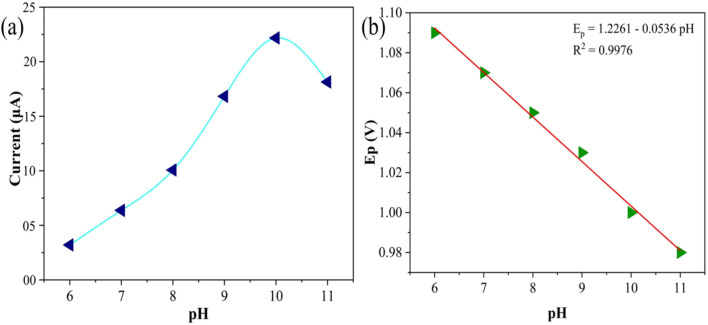
(a) A plot of electrolyte pH *versus* ATN oxidation peak current at a scan rate of 0.05 V s^−1^ and (b) a linear plot of pH *versus* ATN oxidation peak potential.

The influence of electrolyte pH on the electrochemical oxidation of ATN was systematically investigated through cyclic voltammetry, focusing on its effect on both the redox behavior of ATN and its interaction with the SPION-15%AC/GCE surface. The study encompassed pH values ranging from 6 to 11, revealing significant pH-dependent variations in the cyclic voltammograms. Among the tested conditions (pH 6, 7, 8, 9, 10, and 11), the most favorable CV profile—characterized by the highest peak current and the lowest oxidation potential (*E*_p_) was observed at pH 10. The correlation between ATN oxidation peak currents and electrolyte pH is presented in Fig. 10(a). Furthermore, electrolyte pH significantly influenced the oxidation potential of ATN, directly impacting the electron transfer kinetics and overall electrochemical efficiency. A linear relationship was observed between the oxidation potential of ATN and the pH of the electrolyte (Fig. 10(b)) and is expressed using the following regression equation:

7 *E*_p_ (V) = 1.2261 − 0.0536 pH (*R*^2^ = 0.9976)

The slope of 0.0536 V per pH closely aligns with the theoretical Nernstian value, suggesting a reaction mechanism involving an equal number of protons and electrons during the oxidation process. This pH dependency underscores the role of proton-coupled electron transfer in the electrochemical behavior of ATN.

### 3.3. Quantification of ATN using DPV analysis and its detection in real samples

#### 3.3.1. Linear dynamic range, detection limit and sensitivity

**Fig. 11:**
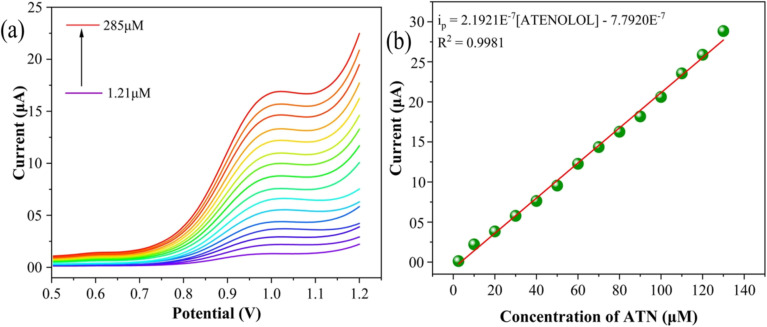
(a) Voltammograms representing the DPV curves for ATN oxidation at the SPION-15%AC/GCE surface in BR buffer solution (pH 10) at a scan rate of 0.05 V s^−1^, (b) calibration plot for the DPV curves under the same experimental conditions.

Differential pulse voltammetry (DPV) is widely recognized for its ability to minimize background current, ensuring precise and reproducible measurements. The critical parameters that establish the efficacy of the proposed ATN sensor – namely, sensitivity, linear dynamic range, limit of detection (LOD), and limit of quantitation (LOQ) – were evaluated using this technique. To develop a rapid and highly sensitive platform for ATN detection and quantification, DPV was employed to measure the current response at the SPION-15%AC/GCE surface over varying ATN concentrations. As depicted in Fig. 11(a), a distinct and linear increase in the oxidation current was observed within the concentration range of 1.21 μM to 285 μM. The SPION-15%AC/GCE sensor demonstrated exceptional performance, achieving a regression coefficient of 0.9981. The calibration curve, shown in Fig. 11(b), provided the following linear equation:

8 *i*_p_ = 2.1921 × 10^−7^[ATN] − 7.7920 × 10^−6^ (*R*^2^ = 0.9981)

Using the slope value (*S*) of the calibration curve, the LOD and LOQ were calculated using the equations (3.3*σ*/*S*) and (10*σ*/*S*), where *σ* represents the standard deviation of the current response. The LOD and LOQ for ATN detection were determined to be 0.401 μM and 1.21 μM, respectively. This remarkable micromolar sensitivity highlights the superior analytical performance of the SPION-15%AC/GCE sensor, attributed to its streamlined fabrication process that avoids the need for complex modifications. A comparative analysis of the SPION-15%AC/GCE sensor's performance for ATN oxidation is summarized in Table 1, further emphasizing its potential for practical applications in electrochemical sensing.

**Table 1:** Comparison of the obtained results with the reported literature

Sl no.	Measurement method	Electrode modification	LOD (μM)	Linear concentration range	Ref.
1	DPV	Fe_2_O_3_–MCM-41–nPrNH2–CPE	2.1	8–205 μM	25
2	DPV	Zn-GO/GCE	2.5	7.8–84 μM	3
				167–639 μM	
3	DPV	GNPs/MWCNTs/GCE	0.5	1–60 μM	4
4	DPV	MgO/SPE	1.76	6.66–909.09 μM	26
5	DPV	GN-CPE	0.073	0.990 μM–0.167 mM	43
6	DPV	HgS/graphene/GCE	0.05	0.5–50.0 μM	44
7	**DPV**	**SPION-15%AC/GCE**	**0.401**	**1.21–285 μM**	**This work**

#### 3.3.2. Storage stability, selectivity, reproducibility and repeatability studies

**Fig. 12:**
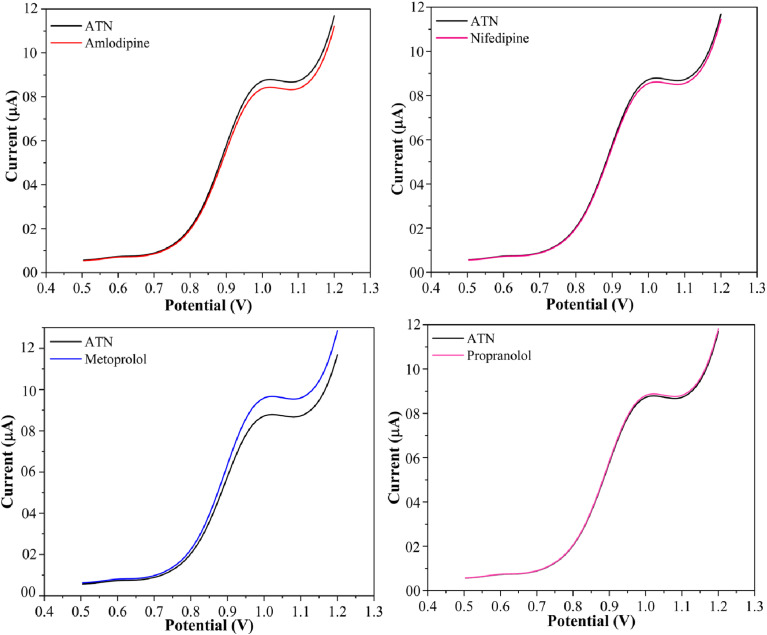
Interference studies of SPION-15%AC/GCE for 80 μM ATN and 800 μM amlodipine besylate, nifedipine, metoprolol and propranolol.

The selectivity of an electrochemical sensor is a crucial factor in its development. To evaluate potential interference, studies were conducted using SPION-15%AC/GCE in the presence of a few commonly prescribed antihypertensive drugs in ATN co-formulated pharmaceutical preparations. These included active ingredients frequently combined with ATN, such as amlodipine, nifedipine, metoprolol, and propranolol. DPV was performed with an ATN concentration of 80 μM, alongside an 800 μM excess of these interfering compounds. As illustrated in Fig. 12, the DPV results demonstrated that even at significantly higher concentrations, amlodipine, nifedipine, and propranolol in a Britton–Robinson buffer solution (pH 10) caused no substantial alteration in the peak current of ATN. A significant rise in the oxidation peak current of ATN was observed in the presence of metoprolol at higher concentrations. However, when ATN and metoprolol were present in equal concentrations, no interference was detected. This highlights the sensor's robust selectivity in the presence of common pharmaceutical additives.

**Fig. 13:**
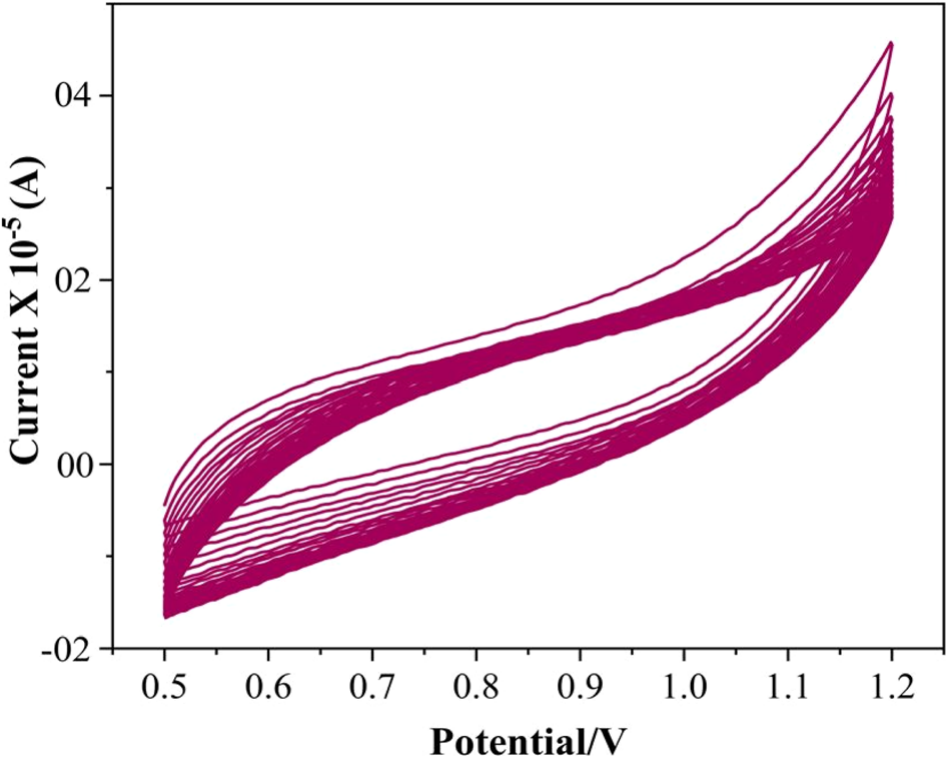
Cyclic voltammogram of SPION-15%AC/GCE at the scan rate of 0.05 V s^−1^ in 0.04 M BR buffer (pH 10), run continuously for 25 cycles.

To evaluate the reproducibility of the proposed method, five independent sets of SPION-15%AC/GCEs were fabricated under identical conditions and tested for their response to 80 μM ATN. The results revealed a remarkable relative standard deviation of only 1.5%, highlighting the method's reproducibility. Furthermore, the SPION-15%AC/GCE demonstrated excellent durability, retaining 96.3% of its original response after being exposed to air for 10 days and undergoing eight successive trials. This performance reflects the sensor's exceptional storage stability and repeatability. The stability of the electrode was further confirmed through 25 continuous cyclic voltammetry cycles in BR buffer without ATN (illustrated in Fig. 13). Impressively, the current response exhibited a negligible decline of less than 2.8% between the initial and final cycles. These results underline the sensor's robustness and long-term reliability, showcasing its potential for sustained and dependable performance across multiple uses and extended periods of exposure.

#### 3.3.3. Detection of ATN in dosage forms

The proposed method was effectively applied to determine ATN in commercially available tablet formulations using the SPION-15%AC/GCE and the DPV technique. Through the standard addition method, a prominent oxidation peak for ATN was detected around 0.97 V in the DPV curves obtained from real sample analyses. The results, detailed in Table 2, underscore the superior efficiency of the developed approach. With recovery rates ranging between 99.56% and 100.2%, the method demonstrates remarkable precision and reliability, making it a highly dependable tool for pharmaceutical analysis.

**Table 2:** Detection and quantification of ATN in dosage forms

Sample	Sample no.	Amount spiked (μM)	ATN expected (μM)	ATN found^a^ (μM)	Recovery %	Relative error	RSD
Ziblok tablets	1	0.0	—	149.5	—	—	1.00
	2	5.0	154.5	153.1	99.74	0.26	1.05
	3	10.0	159.5	160.1	100.2	0.21	0.98
Aten heal tablets	4	0.0	—	199.8	—	—	1.05
	5	5.0	204.8	204.6	99.82	0.18	1.13
	6	10.0	214.8	213.9	99.79	0.18	1.02
Hipres 50 tablet	7	0.0	—	248.9	—	—	1.00
	8	5.0	253.9	253.7	99.87	0.12	0.97
	9	10.0	263.9	262.8	99.56	0.13	1.18

a The mean of three estimations.

## 4. Conclusion

In conclusion, this study successfully developed a rapid and efficient electrochemical sensor for atenolol (ATN) detection, utilizing a glassy carbon electrode (GCE) modified with SPION-AC nanocomposites. The synergistic effect between SPIONs and activated carbon (AC) significantly enhanced the sensor's electrochemical performance, with the SPION-15%AC composite demonstrating optimal sensitivity. This innovative platform achieved a broad linear detection range (1.21–285 μM) and an impressive detection limit of 0.401 μM, showcasing excellent selectivity even amidst high-concentration interferents. The sensor exhibited outstanding reproducibility, stability, and practical applicability, as validated by its successful performance in spiked commercial tablet samples. These findings highlight the potential of SPION-AC nanocomposites in advancing precise, sensitive, and versatile drug detection technologies, paving the way for improved patient monitoring and therapeutic management.

## Data availability

The data supporting this article have been included in the references section of the manuscript and have been cited accordingly in the text.

## Conflicts of interest

There are no conflicts to declare.
